# Uniting Councils on Aging and Research Teams: A Collaborative Approach to Brain Health and Dementia Prevention

**DOI:** 10.1093/geroni/igaf122.856

**Published:** 2025-12-31

**Authors:** Ryan Mace, Makenna Law, Elizabeth Connell, Madeline Noonan, Corinne White, Ana-Maria Vranceanu

**Affiliations:** Massachusetts General Hospital, Boston, Massachusetts, United States; Massachusetts General Hospital, Boston, Massachusetts, United States; Massachusetts Councils on Aging, Franklin, Massachusetts, United States; Massachusetts Councils on Aging, Franklin, Massachusetts, United States; Age Strong Commission, Boston, Massachusetts, United States; Massachusetts General Hospital, Boston, Massachusetts, United States

## Abstract

Older adults with subjective cognitive decline (SCD) face elevated dementia risk, yet preventive lifestyle trials frequently encounter enrollment barriers, including low awareness, limited trust, and accessibility challenges. Senior centers are ideal partners to address these barriers, given their established roles as trusted community hubs offering health promotion, social engagement, and educational programs for older adults. My Healthy Brain (MHB), an NIA K23 Stage 1b RCT, evaluated the feasibility of a mindfulness-based group intervention targeting lifestyle dementia risk factors for older adults with SCD. MHB collaborated with +350 senior centers through the Massachusetts Councils on Aging (MCOA) to enhance recruitment, diversify enrollment, and improve intervention accessibility. Engagement strategies included co-creation of trial procedures with MCOA leadership and local senior center staff through planning meetings and mentor involvement; community brain health presentations (11 events; 22 hours total); early ethics and regulatory approval from the community partner; presence at community events without enrollment pressure; and recruiting through local champions and peer ambassadors. Inclusive practices involved parallel clinical groups for individuals ineligible for trial participation and a participant “reunion” post-trial for feedback and dissemination. MCOA-affiliated channels generated over one-third (35%) of trial enrollment. All enrolled participants (N = 60) completed the intervention, demonstrating high feasibility and acceptability. The community-academic partnership enhanced enrollment diversity, improved community trust, and catalyzed new initiatives (e.g., PCORI submission to reduce loneliness in underserved older adults). This collaboration advances a concept-to-­completion framework for dementia prevention trials, offering a replicable and sustainable blueprint for engaging community organizations and older adults in gerontological research.

